# Genetic Editing of HBV DNA by Monodomain Human APOBEC3 Cytidine Deaminases and the Recombinant Nature of APOBEC3G

**DOI:** 10.1371/journal.pone.0004277

**Published:** 2009-01-26

**Authors:** Michel Henry, Denise Guétard, Rodolphe Suspène, Christophe Rusniok, Simon Wain-Hobson, Jean-Pierre Vartanian

**Affiliations:** 1 Molecular Retrovirology Unit, CNRS URA 3015, Institut Pasteur, Paris, France; 2 Biology of Intracellular Bacteria Unit, Institut Pasteur, Paris, France; Institut Pasteur Korea, Republic of Korea

## Abstract

Hepatitis B virus (HBV) DNA is vulnerable to editing by human cytidine deaminases of the APOBEC3 (A3A-H) family albeit to much lower levels than HIV cDNA. We have analyzed and compared HBV editing by all seven enzymes in a quail cell line that does not produce any endogenous DNA cytidine deaminase activity. Using 3DPCR it was possible to show that all but A3DE were able to deaminate HBV DNA at levels from 10^−2^ to 10^−5^ *in vitro*, with A3A proving to be the most efficient editor. The amino terminal domain of A3G alone was completely devoid of deaminase activity to within the sensitivity of 3DPCR (∼10^−4^ to 10^−5^). Detailed analysis of the dinucleotide editing context showed that only A3G and A3H have strong preferences, notably CpC and TpC. A phylogenic analysis of A3 exons revealed that A3G is in fact a chimera with the first two exons being derived from the A3F gene. This might allow co-expression of the two genes that are able to restrict HIV-1Δ*vif* efficiently.

## Introduction

The human APOBEC3 locus on chromosome 22q13.1 encodes at least seven genes encoding cytidine deaminases with single stranded DNA substrate specificity [1]. While their precise cellular roles remain to be defined, human APOBEC3F and 3G (A3F and A3G) are so active on nascent lentiviral cDNA that these retroviruses have evolved a discrete gene product, the Vif protein, to counter their otherwise devastating effects [2]–[9]. At much reduced frequencies, human APOBEC3 (A3) enzymes can hyperedit HBV genomes as well as those of classical retroviruses such as spumaviruses and human T cell leukaemia virus type 1 *in vitro* [10]–[15]. Other A3 members restrict the transposition of the LINEs and Alu retroelements when over-expressed in transfection assays [16]–[21]. The expanded 7 gene A3 locus is, for the moment, a singular feature of macaque, chimpanzee and human genomes. For the rodent, pig, cattle, cat, dog and horse genomes there are from 1 to 6 APOBEC3 homologues, some of which can be coupled by intergenic splicing [22]–[24].

Three APOBEC3 enzymes (A3A, A3C & A3H) encode a cytidine deaminase domain (CDD) containing a zinc finger motif, while the remaining four (A3B, A3DE, A3F and A3G) encode two CDDs. All CDDs are characterized by the highly conserved HXEX_n_PCX_2–4_C motif that chelates the zinc atom. The glutamic acid residue (E) is considered to potentiate a water molecule that acts as the nucleophile in the oxidation of the C4 amino-group [25]–[28]. Despite this hallmark, not all CDDs bearing this motif are active as judged by phenotypic assays [29]. Exchanging CDDs between A3F and A3G, as well as mutational analyses, have shown that cytidine deaminase activity essentially resides within the second, or carboxy-terminal domain, or CDD2 [29]–[31]. A recent study has shown that both CDD1 and CDD2 of the arteriodactyl APOBEC3F and A3G are functional [22].

Despite many reports of APOBEC editing of viral genomes in experimental systems, only for the lentiviruses, Friend murine leukaemia virus and hepatitis B virus (HBV) have hyperedited genomes been described *in vivo* [12], [32], [33]. The unique *in vivo* HTLV-1 sequence is singular in that it was derived from a squirrel monkey, a New World monkey, experimentally infected by HTLV-1 [34]. At the experimental level, phenotypic assays have been used with great success to identify the effects of APOBEC3 editing on HIV and spumaviruses, although this does not preclude low levels of editing because retrovirus mutation rates are 10–50 fold lower than the error threshold [35]–[37]. In addition, as phenotypic effects have been described in the absence of apparent editing it is not always possible to separate the two components by phenotypic assays [38], [39]. 3DPCR is a method that allows selective amplification of APOBEC edited DNA via modulation of the PCR denaturing temperature [40]. Using this method we have shown that A3B, A3C, A3F and A3G are able to edit a few percent of *de novo* produced HBV genomes *in vitro* [14], [41], [42]. *In vivo* the method was capable of detecting edited HBV genomes at frequencies of ∼10^−4^ [14], [42].

We set out to explore and compare the impact of all seven human APOBEC3 deaminases on the HBV genome. With the sensitivity of 3DPCR it should be possible to identify low levels of editing that might not be readily picked up in phenotypic assays. Using the quail cell line QT6 that does not generate any endogenous editing background, it is shown that 6/7 APOBEC3 enzymes can deaminate a small fraction of hyperedited HBV genomes *in vitro*. CDD1 of A3G is totally devoid of deaminase activity to within the sensitivity of 3DPCR (∼10^−4^). Careful phylogenetic analysis revealed that APOBEC3G is a chimera with the first two exons coming from APOBEC3F.

## Results

### HBV editing by monodomain A3A and A3H

3DPCR is so sensitive that it can amplify heavily APOBEC3 edited HIVΔ*vif* genomes even from a permissive cell line such as 293T [40]. It was presumed that this resulted from clonal heterogeneity whereby a small fraction of cells are expressing high levels of A3F/G. This phenomenon has been encountered for some other cell lines, such as Huh7 (not shown). To overcome this problem we screened a number of avian cell lines because the avian genome does not encode APOBEC1 or APOBEC3 homologues [43]. By contrast, it encodes a single copy of AID and APOBEC2. The quail muscle fibroblast cell line QT6 was chosen for it is readily transfectable.

**Figure 1:**
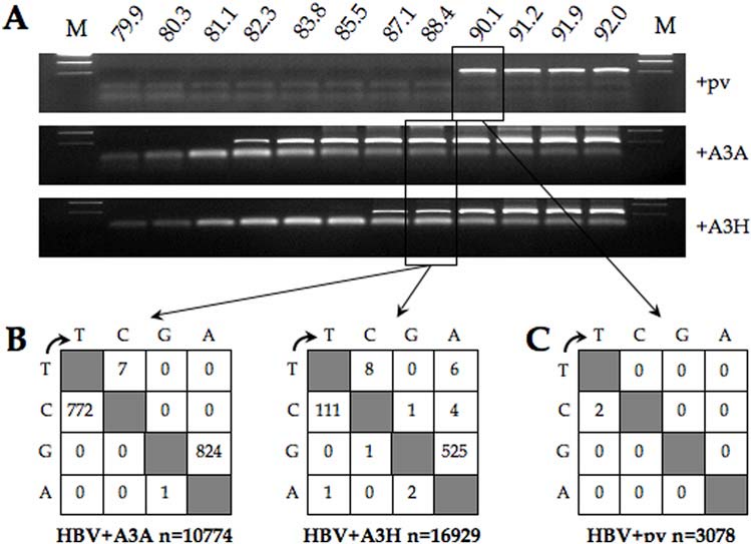
Monodomain A3A and A3H can hyperedit HBV DNA. A) 3DPCR amplification of HBV DNA from on A3A and A3H/pCayw co-transfections of QT6 using a 12°C gradient across the heating block. M denotes molecular weight markers, while pv refers to the empty expression vector. Primer-dimers represent the low molecular weight band. B) Mutation matrices compared to the plus strand as reference where n indicates the number of bases sequenced. C) Mutation matrix for the pCayw plus plasmid vector control.

QT6 cells were transfected with the infectious molecular clone of HBV, pCayw, in the presence or absence of A3A and A3H expression plasmids, the two A3 genes not examined in our previous study [14]. Seventy-two hours post-transfection, supernatant virus was harvested and treated with DNaseI. By decreasing the denaturation temperature (Td) it was shown that ∼90°C was the minimal temperature allowing amplification of HBV infection alone (Figure 1A). By contrast, 3DPCR could recover HBV DNA from the A3A and A3H co-transfections at temperatures as low as 82.3 and 87.1°C respectively.

The 3DPCR products from the Td = 88.4°C amplification were cloned and up to 60 clones sequenced per sample. A complete collection of hypermutated sequences is available in Figures S1. The A3A and A3H enzymes were able to extensively deaminate HBV DNA (Figure 1B). Both DNA strands were heavily edited with mean editing frequencies of 20.5% and 40.1% for A3A on the minus and plus strands respectively, and 6.2% and 11.7% for A3H. All sequences were unique indicating that they represented independent molecular events. Indeed, the degree of A3A editing was among the most extensive we have observed using HBV– up to 95% of target C residues were edited whether they be on the plus of minus strands (Figure S1).

3DPCR products from the last positive amplification (Td = 90.1°C, Figure 1A) for the HBV alone control were also cloned and sequenced. No hypermutated sequences were identified at all indicating that the QT6 cell line did not generate a background editing signal (Figure 1C). To confirm this, a HIVΔ*vif* molecular clone was transfected into QT6 cells and the resulting progeny used to infect the CEMX174 cell line. A 3DPCR cloning and sequencing analysis failed to identify any viral hypermutants (not shown), supporting the conclusion that the QT6 cell line does not generate any background signal.

### Uniquely the C-domain of APOBEC3G is active

**Figure 2:**
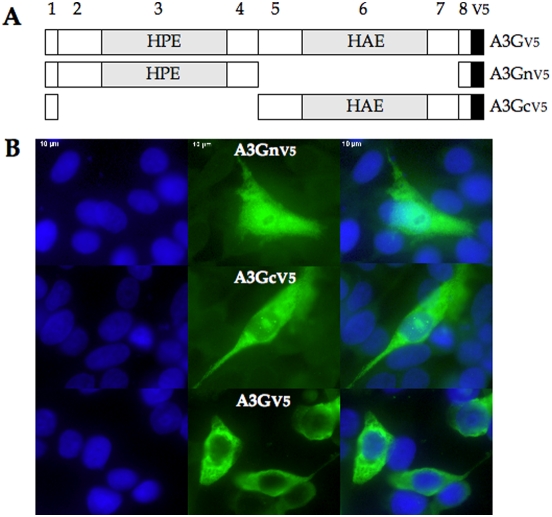
Monodomain variants of A3G are stably expressed. A) Boxes denote each of the eight exons; the shaded 6^th^ exon harbours the zinc finger motif, HAEX_n_PCX_2_C. Shaded exon 3 encodes the singular HPEX_n_PCX_2_C variant. The V5 tag is shown as a black box. B) Confocal immunofluorescence of the three constructs expressed in QT6 cells. DAPI stained cells are on the right, V5 tagged constructs were revealed with x-labelled monoclonal to the V5 tag (centre), while the merged photos are given to the right.

Although the literature indicates that the amino-terminal domain of A3G is without editing activity [30], [31], this doesn't formally preclude a weak-editing activity. As 3DPCR has a greater dynamic range compared to phenotypic assays, 4 logs compared to ≤1.3 logs, we addressed the question of the activity of A3G CDD1 and CDD2 by two approaches. Individual A3G N- and C-terminal domains (CDD1 and CDD2 respectively) genes were made by deletion of exons 5–7 and 2–4 respectively compared to the parental molecule generating A3Gn and A3Gc respectively (Figure 2A). These constructs differ subtly to others [30], [31], [44] in that the core exons 2–4 or 5–7 are always surrounded by exons 1 and 8. Following transfection of HeLa cells the stability of the A3Gn and A3Gc was analyzed by immunofluorescence microscopy using FITC-labelled anti-V5 antibodies. Both constructs were as stable as the parental A3G. Although A3Gn showed a somewhat greater nuclear distribution compared to A3G (Figure 2B), a substantial fraction was in cytoplasm, which is where HBV capsid assembly occurs.

**Figure 3:**
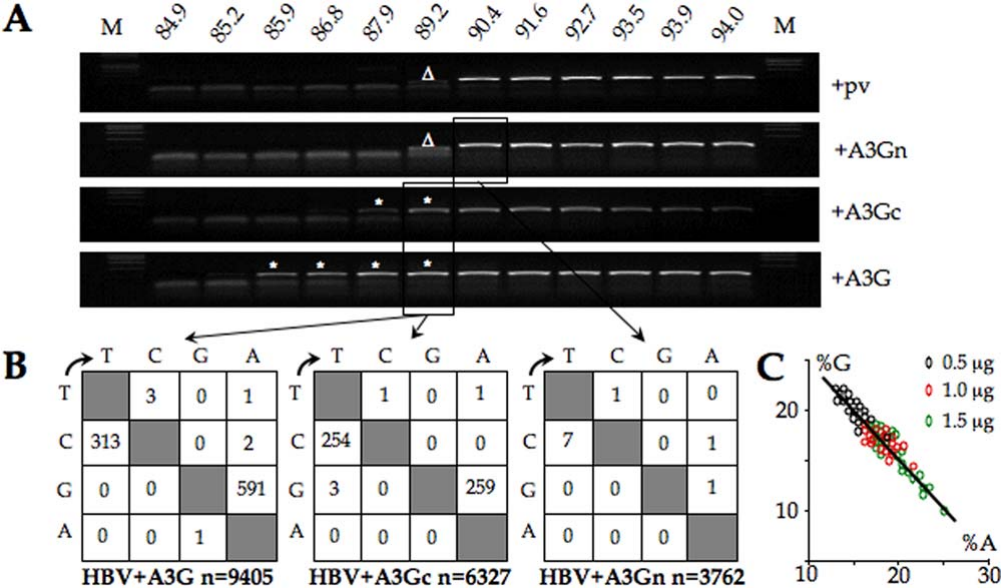
Uniquely the C-terminal domain of A3G is catalytically active. A) 3DPCR amplification using an 11°C gradient in denaturation temperature across the heating block. M denotes molecular weight markers, pv the empty expression vector, the white asterisks denote hyperedited HBV DNA, while the white triangles indicate PCR products containing internal deletions (confirmed by sequencing of cloned products). B) Mutation matrices for A3G and A3Gc on QT6 cells where n indicates the number of bases sequenced. C) The inter-conversion of G→A is related to the concentration of A3G co-transfected.

QT6 cells were transfected by pCayw in the presence or absence of the different expression plasmids (A3G, A3Gn, A3Gc). Seventy-two hours post-transfection, virus was harvested and treated with DNaseI. At 90.4°C it was possible to amplify the complete HBV segment alone (Figure 3A). At 89.2°C lower molecular weight bands were found for the negative control and A3Gn (Δ, Figure 3A), however cloning and sequencing revealed that these sequences contained internal deletions and no C→T transitions. By contrast, DNA was recovered between 87.9–89.2°C for the A3Gc transfection and 85.9–89.2°C for the A3G control (*, Figure 3A).

Cloning and sequencing of the 3DPCR products confirmed that these sequences were indeed hypermutated (Figure 3B, Figure S1). The average editing efficiencies for genomes recovered at the same denaturation temperature (89.2°C) were comparable for A3G (G→A 14.9%; C→T 21.7%) and A3Gc (G→A 9.5%; C→T 22.6%) indicating that the carboxy-terminal domain alone contains the editing function. To see whether the protocol could pick up differing degrees of editing a titration was performed using A3G. As can be seen form Figure 3C, the degree of editing is in proportion to the mean A3G plasmid concentration. Accordingly, it may be presumed that the 3DPCR products recovered for A3G down to 85.9°C are even more hypermutated.

**Figure 4:**
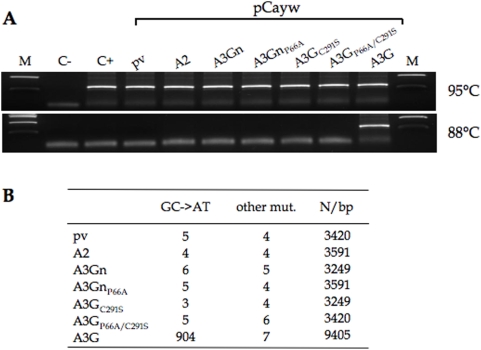
Proline 66 is not responsible for the inactive N-terminal Zn finger domain of A3G. A) PCR amplification of HBV DNA from a variety of co-transfection experiments at 95°C and the restrictive temperature of 88°C. M denotes molecular weight markers; C-, amplification control without DNA; C+, pCayw alone; pv the empty expression vector as negative control; A2, APOBEC2 as another negative control. Primer-dimers represent the low molecular weight band. B) Mutational analysis of the cloned 95°C PCR products and those of the positive A3G control at 88°C.

To explore the hypothesis that CDD1 of A3G was slightly active uniquely in the presence of CDD2, a C291S mutant of A3G was used [15]. Cysteine 291 is one of the residues that chelates the zinc atom in CDD2 and is essential to the activity of all A3 enzymes tested to date. HBV+A3G_C291S_ were transfected into QT6 cells and 3DPCR performed on supernatant virus. As can be seen from Figure 4A no products were recovered at the restrictive temperature of 88°C. Products recovered at 95°C amplifications were cloned and sequenced. No excess of GC→AT substitutions was noted compared to controls (Figure 4B).

Among all human APOBEC3 molecules, CDD1 of A3G is unique in that the alanine residue in the highly conserved HAEX_n_PCX_2_C motif is substituted by proline, a feature characteristic of the primate A3G lineage back to macaque and baboons [45]. Given the profound changes a proline residue can have on protein folding, a P→A substitution was engineered into codon 66 of the A3Gn and A3G_C291S_ constructs. This P66A substitution failed to recover any deaminase activity in either a mono- of double domain construct (A3Gn_P66A_ and A3G_P66A/C291S_, Figure 4).

### Comparable A3G and A3Gc hot and cold spots

**Figure 5:**
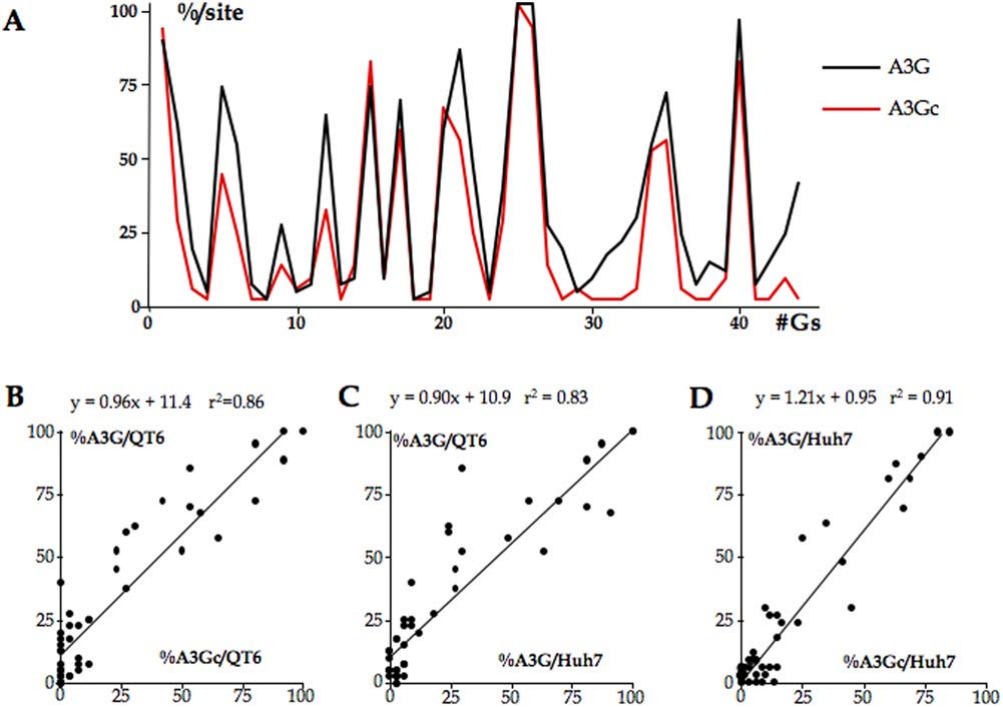
Human A3G and A3Gc hot and cold spots are highly correlated. A) Individual editing frequencies across the minus strand target cytidine residues for A3G and A3Gc co-transfections of Huh7 cells. These represent the fraction of A3 edited sequences bearing an edited C at a given position. B–D) Highly correlated site-specific editing frequencies for A3G, A3Gc/HBV co-transfections of QT6 and Huh7 cells. The site-specific editing frequencies calculated for a given data set are correlated with the site-specific editing frequencies from another data set at the level of each C residue. The identities of the data sets are given on the x and y-axes. Least-mean squared fits to the data are also given.

The editing frequencies of all 45 cytidine targets in minus strand HBV DNA for A3G and A3Gc in the QT6 derived experiments were compared and found to be highly correlated (Figure 5A, B), again indicating that editing specificity resided in CDD2. In order to see if the cell line used in the transfection essay could influence the site specific editing frequencies, A3G and A3Gc editing of HBV was compared in both cell lines QT6 and Huh7. Site specific editing frequencies were highly correlated between the two cell lines for both APOBEC3 constructs (Figure 5C, D) indicating that the QT6 cell line did not introduce any bias into the editing process, which suggests that editing occurs independently of any human protein partner.

To estimate the frequency of editing, limiting dilution of first round PCR products was performed at 95°C and the restricting temperature of 89.2°C. The frequencies of hyperedited genomes were A3A≥10^−2^, A3G≥10^−3^, A3Gc≥10^−4^ and A3H≥10^−5^ (data not shown). The values are lower limits in that not all the cells were doubly transfected by pCayw and the A3 expression vector. The results show that the A3Gn domain increases the efficiency of A3G editing, while A3A turns out to be particularly efficient (Figures 1A and 3A).

### Dinucleotide context of HBV editing by six A3s

**Figure 6:**
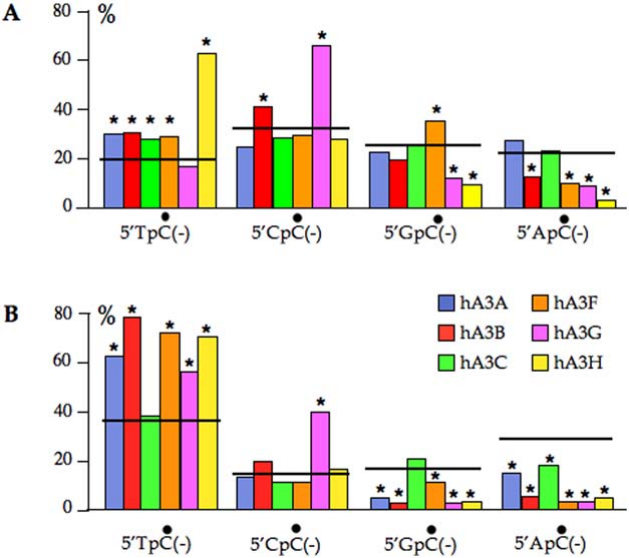
Cytidine deamination dinucleotide context varies as a function of the target sequence. A) Cytidine editing of the minus DNA strand of HBV by six A3s, the horizontal line indicating the expected values based on the sequence composition of the target. B) Cytidine editing of the minus DNA strand of HIV by six A3s, the horizontal line indicating the expected values based on the sequence composition of the target. Asterisks denote statistically significant deviations from the expected value (p<0.001).

As there was no difference between site specific A3G editing of HBV in the QT6 and Huh7 cell lines, the dinucleotide contexts for G→A hypermutants can be compared using data sets for A3A and A3H derived from QT6 and A3B, A3C, A3F ad A3G derived from Huh7. As mentioned above, we were unable to recover any A3DE hyperedited HBV genomes. In view of the general excess of G→A hypermutants over C→T hypermutants, only the former, which reflect editing of the minus strand were considered. Sequences with >80% of cytidine residues edited were excluded from the analysis in order not to mask any dinucleotide preferences. Obviously, the greater the degree of editing, the more the observed and expected dinucleotide frequencies converge. Three distinct patterns were apparent. A3G showed a strong and significant preference for CpC while A3H showed a predilection for TpC (Figure 6A). Both showed a strong aversion for GpC and ApC. The remaining four A3s showed weaker dinucleotide biases with A3F even showing a slight, but significant bias in favour of GpC.

**Figure 7:**
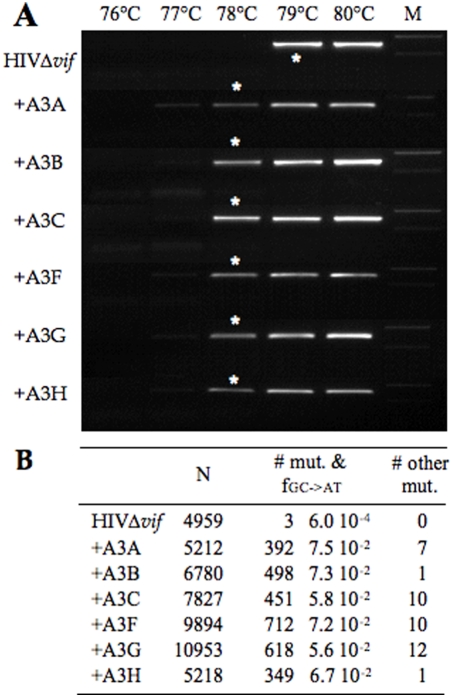
HIV-1Δ*vif* editing by 6 A3 deaminases. A) 3DPCR of cDNA harvested 18 h post-infection of CEMx174 cells. Virus stocks were made using QT6 cells, white asterisks represent the PCR product cloned and sequenced. B) Mutation statistics for the HIV-1 hypermutants.

The lack of any strong preference for TpC by A3F was surprising as it is a frequent finding in the HIV-1 literature [4], [8], [46]–[48]. In view of this, we generated comparable hyperedited data sets for HIVΔ*vif* and the same 6 A3 constructs co-transfected into QT6 cells. Supernatant HIV was then used to infect CEMX174 cell line. DNA was recovered after 18 h and 3DPCR performed on a region corresponding to the hypervariable V1V2 regions of *env*, the restrictive temperature being 78°C (Figure 7A). 3DPCR products amplified at 78°C were cloned and sequenced. Large numbers of hypermutants were obtained all with G→A mutation frequencies >100 in excess of the control (Figure 7B, Figure S2). The dinucleotide frequencies associated with deamination on the minus strand (G→A on the plus strand) were analyzed in a comparable manner to the HBV hypermutants, that is those with >80% editing were eliminated from Figure 6B. Five of the six A3s showed a strong and significant preference for TpC, while only A3G showed any specificity for CpC indicating that dinucleotide preferences may result from the presence of hot spots within a target sequence (Figure 6B). This time, A3F showed an aversion for GpC, unlike its behaviour on a HBV template (Figure 6A). Nonetheless, the preference of A3G for CpC and A3H for TpC remained.

Human A3C showed the least polarized dinucleotide editing preference on either an HBV of HIV template. The A3C sequence is unique in encoding a serine at position 188 as opposed to an isoleucine residue conserved among all other A3 deaminases, APOBEC2, AID and even APOBEC1 [1]. A S188I mutant of the A3C-V5 tagged construct was generated and found to deaminate HBV in an indistinguishable manner (frequency, dinucleotide context) compared to the parental sequence without (data not shown).

### Exon based phylogenies for A3s

**Figure 8:**
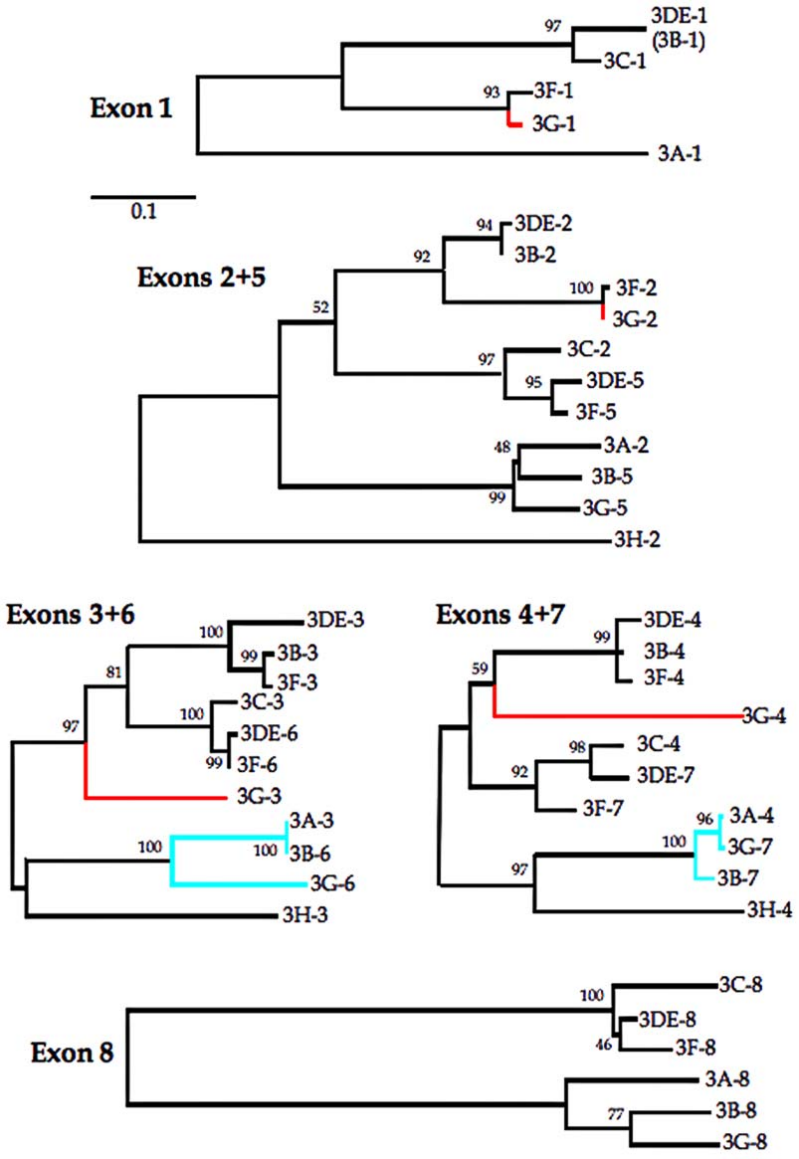
Phylogenic analysis of A3 exons indicates past intergenic recombination. All Neighbor-Joining trees are drawn to the same scale. Numbers at branching nodes are percentages of 1000 bootstrap replications - only bootstrap values >40 are given. A3H was excluded was excluded from the exon 1 and 8 trees for lack of significant homology. A3B was excluded from the exon 1 tree because it is only 71 bases long and led to an even less robust tree. Nonetheless, as it differs from the A3DE sequence by only 2 bases, it is shown in parentheses. The anomalous A3G sequences are highlighted in red. Another case of possible recombination is highlighted in sky blue as the differing (3A-3, 3B-6, 3G-6) and (3A-4, 3G-7) 3B-7) topologies are supported by strong bootstrap values. After gap stripping, the lengths of each alignment were as follows: exon 1, 89 bp; exons 2+5, 135 bp; exons 3+6, 256 bp; exons 4+7, 113 bp and exon 8, 240 bp.

The modular nature of the human A3s as well as extant phylogenic studies have shown that recombination can occur between CDD1 and 2 [49]. As the exon/intron structures for CDD1 and CDD2 are strictly comparable there is the possibility of recombination between exons within CDDs. An exon-based phylogenic analysis showed that the mainly non-coding exons 1 and 8 are, perhaps not surprisingly, fixing mutations faster than the coding exons 2+5, 3+6 and 4+7, which themselves showed a generally congruent topology (Figure 8). The obvious exception is A3G shown in red. Here, exons 1+2 strongly cluster with those of A3F. As A3F exons 1, 2, 3 and 4 always cluster with those of A3B and A3DE it would appear that A3G exon 2 is anomalous. Indeed the coding regions of the first two exons of A3F and A3G differ by 1 base (0.6%, or a single amino acid residue Δ∼2%) while exons 3 and 4 differ by 121 nucleotides (∼30%, or 62 amino acid residues, Δ∼47%) and 2 indels. The 5′ breakpoint may map as far as 6.5 kb upstream of exon 1 (not shown). This event seems to exist as far back as the *Macaca* and *Cercopithicus* monkey lineages (see Supplementary table 3 in [50]).

## Discussion

HBV is sensitive to low frequency editing by 6/7 human APOBEC3 cytidine deaminases. The finding of A3A hyperedited HBV genomes is novel and contrasts with a negative report [51]. Those for A3H confirm a recent study [24] and show that all the monodomain A3 enzymes are able to edit HBV and HIV cDNA. Indeed, A3A and A3H are some of the most efficient cytidine deaminases that we have worked with [24]. As the hypermutant frequencies were in the ∼10^−2^ to 10^−5^ range, deamination may represent an insufficient selection pressure to cause the virus to adapt, unlike the lentiviruses that evolved a *vif* gene. While APOBEC3s can be found in the viral HBcAg capsid [14], [15], packaging is inefficient, certainly with respect to the HIV-1Δ*vif*/A3G as reference. This follows from a consideration of the intravirion enzyme concentration: the inner HBcAg core volume is ∼9 zl [52]. Ignoring the excluded volume due to the HBV genome, a single molecule of any packaged A3 deaminase translates into [A3]>200 µM. Given that A3G hyperediting of ssDNA (f_C→T_∼0.2, [53]) was observed *in vitro* with a enzyme concentration of ∼2 µM, once even a single molecule is packaged within the HBcAg capsid, hyperediting is inevitable. Accordingly, packaging must be inefficient.

A similar argument goes for HIV where the core volume is ∼90 zl [54]. Ignoring the excluded volume of 2 genomic RNAs, one molecule of A3G translates into an intravirion [A3G]>18 µM. As Pathak et al. estimated that on average there are 7 A3G molecules per HIV1Δ*vif* virion when the virus is produced on PBMCs [55], the physiological concentration is [A3G]>130 µM. Unlike HBV, A3F and A3G are efficiently packaged by a HIVΔ*vif* virus meaning that hypermutants may be identified without recourse to 3DPCR.

Genetic editing of HBV is a feature of both the mono- and double CDD APOBEC3s. CDD1 of A3G is devoid of any cytidine deaminase activity (Figure 2) down to the 10^−4^ to 10^−5^ detection level characteristic of 3DPCR {Suspene, 2005 #332; Susp,} and supports the conclusions based on phenotypic analyses *in vitro* or in a mouse model [33]. This feature is not conferred by the evolutionarily conserved substitution of alanine 66 for proline (Figure 4). Given that CDD2 alone contains the same editing specificity as the parental A3G molecule, the role/function of CDD1 remains elusive. Nonetheless, A3G edits HBV cDNA at a higher frequency compared to A3Gc, an observation that concords with a higher editing processivity [56], both demonstrate that CDD1 modulates the function of CDD2. The conservation of the HXEX_n_PCX_2–4_C motif in CDD1 in the absence of demonstrable deamination remains a puzzle.

That six A3 enzymes expressed in QT6 cells are functional suggests that they do not require human protein partners, unless of course it was a highly conserved protein between humans and birds. This conclusion is echoed by *in vitro* experiments where hyperedited DNA was recovered reactions using ssDNA and baculovirus produced A3G [53], while neo-synthesized A3G in low molecular weight form, as opposed to that in a high molecular weight complex, is active on HIV [57], [58]. In addition, when expressed in *E. coli* hA1, A3G and human AID behave as mutators generating C→T transitions [2], [59]. Taken together, these findings indicate that A3 enzymes alone are sufficient to undertake hyperdeamination.

When editing HBV cDNA, only A3G (double domain) and A3H (mono-domain) showed any strong dinucleotide specificity (Figure 6A). By contrast the HIV/A3 literature describes A3F as having a bias for the TpC dinucleotide (Figure 6B and [4], [8], [46]–[48]). The dinucleotide composition of the minus strand of the HBV segment analysed is 20.5% TpC, 31.9% CpC, 25.0% GpC and 22.7% ApC. By comparison, that for the HIV minus strand is even more polarized with 38% TpC, 16% CpC, 18% GpC and 28% ApC. It is possible that the HIV locus, with a greater proportion of TpC encodes a few hotspots allowing A3A, A3B, A3F and A3G editing at this dinucleotide to become significant.

The mammalian APOBEC3 locus has undergone expansion presumably from a single gene. The presence of 1–6 APOBEC3 genes among rodent, carnivore and perissodactyl lineages, comprising both mono- and double domain molecules, all point towards extensive gene conversion [60], [61]. Given that exon/intron structures are highly comparable among A3 genes there was the possibility of recombination between exons. From a phylogenic consideration of the different exons it would appear that A3G is a chimera of at least three different segments (exons 1+2, exons 3+4 and exons 5–8; Figure 8).

**Figure 9:**
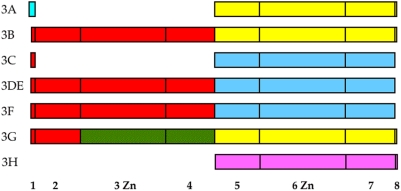
Colour coded representation of A3 coding exons. Colour denotes generally similar overall clustering and hides finer detail. Coding regions are drawn to scale. As the iMet of A3H is within exon 2, exon1 is not shown. As it is not possible in an *a posteriori* analysis to distinguish between recombination and rate heterogeneity, while some clusters are not supported by strong bootstrap values, the schematic should not be overly interpreted.

The ensemble of the A3 phylogenic trees can be resumed in a schematic form where colour reflects the deep phylogenic clustering into three or four groups (Figure 9). Other recombination events are evident, for example, i) the A3A first exon stands out by coding for 10 distinct residues as opposed to 6 highly conserved residues for A3B-G; ii) A3H is unusual in that the first exon is non-coding (see NM_181773); iii) it could be argued that A3C arose from a duplicated A3DE or A3F gene with subsequent deletion of exons 2–4; iv) the discordant arborescence of A3A, A3B and A3G in exons 6 and 7, all of which are supported by good bootstrap values, is tell tale. If true, the latter example would mean that A3G has an even more complex evolutionary history. With this latter example in mind, it is worth noting that the present 7 gene locus in not invariant. A human haplotype dominant in Papua New-Guinea is lacking the A3B gene, while others have cogently argued for loss and gain of the A3H gene [62].

Interestingly, A3F and A3G are precisely the cytidine deaminases that are most active on HIV-1Δ*vif* in phenotypic assays [5], [6], [8], [46]. As the promoters are highly similar, it is expected that their expression would be coordinated and hence they could restrict HIV together, if given the chance. In this context it is worth noting that not only A3F and A3G expression was surprisingly coordinated, the two deaminases formed heterodimers [8]. A3G exon 2 is homologous to exon 5 (Jarmuz). In the recently reported crystal structures for the CCD2 domain of A3G, exon 2 is part of a homodimerization interface (Harris & Goodman). In view of the recombinant nature of A3G, this might contribute to efficient A3F and A3G heterodimer formation.

Two New World monkey (NWM) sequences formally annotated as A3Gs could well be A3F-G chimeras as they were generated by screening genomes with the A3G sequence [50]. As it is a chimera, blast searching alone might well generate a recombinant even though the A3G of the genome in question might not have exons 1+2 akin to A3F. Both of these NWM sequences lack the singular HPE motif in the CDD1 of all other A3Gs. While not definitive, it urges caution. Three gibbon sequences annotated as A3G are clearly A3F-G chimeras – they were compiled by screening of genomic DNA while the break points map to exon boundaries [63]. That three subspecies of gibbon ape each harbour a different A3G chimera is not impossible, although parsimony suggests this to be implausible.

Given the extensive gene conversion and near identity of some exons, for example A3A-4 and A3G-7 (Figure 8), it is understandable that computer programs are unable to always identify correctly an APOBEC3 gene. The above examples are not, unfortunately unique. For example, the *Macaca mulatta* entry XM_001094452, ostensibly for A3G, actually encodes three CDDs. By comparison with the human A3 sequences, it is trivial to show that CDD1+2 are those of a *bona fide* macaque A3F gene while the third domain corresponds to CDD2 of the macaque A3G gene. The *Macaca mulatta* A3D entry XM_001094328 misidentifies the initiator methionine codon – as the coding region of exon 1 is only 6 amino acid residues long, the program presumably missed it. Consequently the A3D entry starts with a conserved Met residue that is actually within the second exon of the human orthologue. Equivalents for A3A and A3B are not annotated in the macaque genome via a NCBI search although it is trivial to find them with a Blast search. A few analogous errors can be found among the annotated genomic chimpanzee A3 sequences.

Apart from these *in silico* issues, some rather mundane issues come to the fore. Given the near identity of some exons across A3 deaminases, for example A3A exon 4 and A3G exon 7 or A3A exon 3 and A3B exon 6, ambiguities may arise when probing Northern and Southern blots with near-complete cDNAs. The same goes for *in situ* RNA hybridization using complete cDNAs. Polyclonal sera could possibly pick up more than one APOBEC3 protein. Depending on the region targeted, there is even the chance that a monoclonal antibody might show a little cross-reactivity.

The study shows that while six human APOBEC3 deaminases can edit HBV albeit at low frequencies, the degree of nascent cDNA editing can be as great as 90%. Recently the A3A, A3C and A3H deaminases have been shown to be active against human papillomavirus DNA in benign and pre-cancerous lesions [64]. Transcriptionally active HPV genomes are in the form of mini-chromosomes complete with nucleosome [65]. If they are vulnerable to cytidine deamination, say in a subset of cells with over-expressed APOBEC3A/C/H, then it raises the intriguing question as to how chromosomal DNA is protected from these three nuclear APOBEC3 deaminases, which are, when given the chance, hugely efficient at editing ssDNA *in vivo* and *in vitro* (Figures 4 and 6).

Activation induced deaminase (AID), which is to all extents and purposes a human APOBEC3 gene [64], albeit found at ch12p13.31, can be specifically targeted to the IgV gene, although the mechanism is not clear. However, there is an ample literature demonstrating ectopic expression of AID in non-lymphoid tissues such as stomach and liver in the case of chronic HCV associated hepatitis [66]–[70]. Indeed, transgenic mice with AID under the control of a hepatotropic promoter give rise to HCCs, paralleling earlier findings for APOBEC1 transgenic mice [71], [72]. While efficient mutators, especially A3A, their effects are surely countered by highly efficient DNA repair machinery and so it may be extremely difficult to identify hyperedited human genes precisely for this reason. From this perspective, hyperedited HBV or dsDNA virus genomes could be seen as a surrogate marker for these powerful mutators especially when associated with predisposing or pre-neoplastic lesions like cirrhosis and low and high-grade intraepithelial lesions of the cervix.

## Materials and Methods

### Plasmids

Human A3A cDNA was obtained from Bryan Cullen, A3G from Naveenan Navaratnam and A3DE from Michael Malim. The remaining human APOBEC3 cDNAs were obtained from an IMAGE clones cDNA library and were sub-cloned in the pcDNA3.1D/V5-His-TOPO expression vector (Invitrogen) as previously described [14]. Construction of A3Gn (N-terminal region or CDD1) was obtained by deleting exons 5–7 from the A3G clone, while A3Gc (C-terminal region or CDD2) was obtained by deleting exons 2–4 (Figure 2A). Plasmid pCayw allows expression of pre-genomic HBV RNA under control of the powerful cytomegalovirus immediate early promoter (CMV-IE) and was provided by Frank Chisari (Scripps Research Institute). The HIV-1 infectious molecular clone pNL4.3 plasmid has already described [73].

### Cells, transfections and infections

Virus stocks were made following transfection of the quail muscle fibroblast cell line QT6. Briefly, 10^5^ QT6 cells were co-transfected with equal amounts (2 µg) of the pCayw or pNL4.3 and A3 expression plasmids using FuGENE6 (Roche). In both experiments, viruses were harvested 72 hours post-transfection, treated with DNaseI (10 U/mL, 1 mM MgCl_2_, 1 h/37°C). HIV virus stock was titrated with a p24 Elisa kit (PerkinElmer) and was used to infect H9 cells for 18 h. Total DNA was extracted using the MasterPure™ complete DNA and RNA purification kit (Epicentre). QT6 cells were maintained in HAM's F40 medium, supplemented with 1% chicken serum, 10% FCS, 5% tryptose phosphate, 2 mM L-glutamine, 50 U/ml penicillin and 50 µg/ml streptomycin while H9 cells were maintained in DMEM medium supplemented with 2 mM L-glutamine, 50 U/ml penicillin and 50 µg/ml streptomycin.

### Immunofluorescence

HeLa cells were grown to a density of 5×10^5^ cells per dish and transfected with 0.4 µg of pcDNA6-mAPOBEC using FuGENE 6 (Roche). After 48 hours, the cells were washed twice with PBS, fixed for 45 minutes in a 50∶50 methanol/ethanol mix. As primary antibodies, a mouse monoclonal antibody specific for the V5 epitope tag (Invitrogen) was used at a 1∶200 dilution for 1 hour at 37°C. Cells were washed twice with PBS, and fluoresceine isothiocyanate-conjugated anti-mouse antibody was used as second antibody (Sigma) at a dilution 1∶200 for 30 minutes at 37°C. Cells were then washed three times in PBS, then incubated with 0.1 µg/ml of 4′, 6′-diamino-2-phenylindole (DAPI) for 10 min. at room temperature to counter stain nuclei. Immunofluorescence was observed by microscopy (Zeiss).

### PCR amplification, cloning and sequencing

All samples were digested with proteinase K (0.1 mg/ml, Eurobio) in lysis buffer at 65°C for 15 min. Total nucleic acids were extracted using the MasterPure™ complete DNA and RNA purification kit (Epicentre). A fragment just 5′ to the HBV X gene was chosen as described [14]. In order to increase sensitivity and specificity, hot start PCR was performed with primers 5′HBVout and 3′HBVout while primers 5′HBVin and 3′HBVin were used for the second round and were already described [14]. G→A hypermutated genomes were recovered using differential DNA denaturation PCR (3DPCR) in a two round procedure [40]. The first reaction involved standard amplification, the reaction parameters were 95°C for 5 min., followed by 35 cycles (95°C for 30 s., 50°C for 30 s., and 72°C for 1 min.) and finally for 10 min. at 72°C for the first round. Differential amplification occurred in the second round using the equivalent of 0.2 µL of the first round reaction as input. Differential amplification was performed by using an Eppendorf gradient Mastercycler S programmed to generate a 4–12°C gradient in the denaturation temperature. Gradient cycling conditions were, from 93°C to 81°C for 5 min., followed by 35 cycles (93–81°C for 30 s., 50°C for 30 s., and 72°C for 1 min.) and finally 10 min. at 72°C. First round PCR gave a 294 bp product, while second round PCR yielded a 213 bp fragment.

The HIV-1 *env* V1V2 region was amplified by a 3DPCR procedure, the first reaction PCR was amplified using primers SK122out 5′ TACCCACARACCCCAACCCACAA and SK123out, 5′ TTTTAGAATYGYAAAAYYAG (R = A, G and Y = C, T). Parameters were 95°C for 5 min., followed by 35 cycles (95°C for 30 s., 50°C for 30 s., and 72°C for 1 min.) and finally for 10 min. at 72°C. Selective 3DPCR was performed at the second round using primers SK122/Rin 54 AAARCCTAAARCCATRTRTA and SK123/Yin 5′ TAATGTATGGGAATTGGYTTAA with the following conditions: 77°C to 83°C for 5 min., followed by 35 cycles (77–83°C for 30 s., 50°C for 30 s., and 72°C for 1 min.) and finally for 10 min. at 72°C. The last PCR product yielded a 304 bp fragment. PCR products were purified from agarose gels (Qiaex II kit, Qiagen, France) and ligated into the TOPO TA cloning vector (Invitrogen, France). After transformation of Top10 Blue cells, up to 40 clones were picked. Sequencing was performed using BigDye Terminator V1.1 (Applied Biosystems).

Primers used for the construction of A3Gn plasmid were E1 and E4 respectively 5′ CACCATGAAGCCTCACTTCAGAAACA and 5′ GTTTTCCTGATTTCTGAGAATCTCCCCCAGCATGAT where the initiator methionine codon is underlined, while for A3Gc plasmid construction primers used were E5 and E8 respectively, 5′ CACCATGAAGCCTCACTTCAGACACTCGATGGATCCACCCA and 5′ CCATTCTCCAGAATCAGGAAAAC. The buffer conditions for amplifications were 2.5 mM MgCl_2_, 50 mM KCl, 10 mM Tris-HCl pH 8.3, 200 µM of each dNTP, 100 µM of each primer, and 2.5 units of Pfu polymerase (Promega) in a final volume of 50 µL. PCR conditions were: 95°C for 5 min., followed by 20 cycles (95°C for 30 s., 55°C for 30 s., and 72°C for 30 s.) and finally for 10 min. at 72°C. PCR product was purified and cloned in pcDNA3.1D/V5-His-TOPO.

### Phylogenic analyses

The APOBEC3 sequences used in the alignment were: A3A NM_145699; A3B NM_004900; A3C NM_014508; A3DE NM_152426; A3F NM_001006666; A3G NM_021822; and A3H NM_181773. All sequences were aligned using CLUSTALX [74]. Trees were constructed by the Neighbor-Joining method in MEGA (Version 3.1) [75].

## Supporting Information

Figure S1 (0.11 MB RTF)

Figure S2 (0.09 MB RTF)

## References

[1] Jarmuz A, Chester A, Bayliss J, Gisbourne J, Dunham I (2002). An anthropoid-specific locus of orphan C to U RNA-editing enzymes on chromosome 22.. Genomics.

[2] Harris RS, Bishop KN, Sheehy AM, Craig HM, Petersen-Mahrt SK (2003). DNA deamination mediates innate immunity to retroviral infection.. Cell.

[3] Lecossier D, Bouchonnet F, Clavel F, Hance AJ (2003). Hypermutation of HIV-1 DNA in the absence of the Vif protein.. Science.

[4] Liddament MT, Brown WL, Schumacher AJ, Harris RS (2004). APOBEC3F properties and hypermutation preferences indicate activity against HIV-1 in vivo.. Curr Biol.

[5] Mangeat B, Turelli P, Caron G, Friedli M, Perrin L (2003). Broad antiretroviral defence by human APOBEC3G through lethal editing of nascent reverse transcripts.. Nature.

[6] Mariani R, Chen D, Schrofelbauer B, Navarro F, Konig R (2003). Species-specific exclusion of APOBEC3G from HIV-1 virions by Vif.. Cell.

[7] Sheehy AM, Gaddis NC, Choi JD, Malim MH (2002). Isolation of a human gene that inhibits HIV-1 infection and is suppressed by the viral Vif protein.. Nature.

[8] Wiegand HL, Doehle BP, Bogerd HP, Cullen BR (2004). A second human antiretroviral factor, APOBEC3F, is suppressed by the HIV-1 and HIV-2 Vif proteins.. EMBO J.

[9] Zhang H, Yang B, Pomerantz RJ, Zhang C, Arunachalam SC (2003). The cytidine deaminase CEM15 induces hypermutation in newly synthesized HIV-1 DNA.. Nature.

[10] Delebecque F, Suspene R, Calattini S, Casartelli N, Saib A (2006). Restriction of foamy viruses by APOBEC cytidine deaminases.. J Virol.

[11] Lochelt M, Romen F, Bastone P, Muckenfuss H, Kirchner N (2005). The antiretroviral activity of APOBEC3 is inhibited by the foamy virus accessory Bet protein.. Proc Natl Acad Sci USA.

[12] Noguchi C, Ishino H, Tsuge M, Fujimoto Y, Imamura M (2005). G to A hypermutation of hepatitis B virus.. Hepatology.

[13] Rosler C, Kock J, Kann M, Malim MH, Blum HE (2005). APOBEC-mediated interference with hepadnavirus production.. Hepatology.

[14] Suspene R, Guetard D, Henry M, Sommer P, Wain-Hobson S (2005). Extensive editing of both hepatitis B virus DNA strands by APOBEC3 cytidine deaminases in vitro and in vivo.. Proc Natl Acad Sci USA.

[15] Turelli P, Mangeat B, Jost S, Vianin S, Trono D (2004). Inhibition of hepatitis B virus replication by APOBEC3G.. Science.

[16] Bogerd HP, Wiegand HL, Hulme AE, Garcia-Perez JL, O'Shea KS (2006). Cellular inhibitors of long interspersed element 1 and Alu retrotransposition.. Proc Natl Acad Sci USA.

[17] Esnault C, Heidmann O, Delebecque F, Dewannieux M, Ribet D (2005). APOBEC3G cytidine deaminase inhibits retrotransposition of endogenous retroviruses.. Nature.

[18] Jern P, Stoye JP, Coffin JM (2007). Role of APOBEC3 in genetic diversity among endogenous murine leukemia viruses.. PLoS Genet.

[19] Muckenfuss H, Hamdorf M, Held U, Perkovic M, Lower J (2006). APOBEC3 proteins inhibit human LINE-1 retrotransposition.. J Biol Chem.

[20] Schumacher AJ, Hache G, Macduff DA, Brown WL, Harris RS (2008). The DNA deaminase activity of human APOBEC3G is required for Ty1, MusD, and human immunodeficiency virus type 1 restriction.. J Virol.

[21] Schumacher AJ, Nissley DV, Harris RS (2005). APOBEC3G hypermutates genomic DNA and inhibits Ty1 retrotransposition in yeast.. Proc Natl Acad Sci USA.

[22] Jonsson SR, Hache G, Stenglein MD, Fahrenkrug SC, Andresdottir V (2006). Evolutionarily conserved and non-conserved retrovirus restriction activities of artiodactyl APOBEC3F proteins.. Nucleic Acids Res.

[23] Munk C, Beck T, Zielonka J, Hotz-Wagenblatt A, Chareza S (2008). Functions, structure, and read-through alternative splicing of feline APOBEC3 genes.. Genome Biol.

[24] OhAinle M, Kerns JA, Li MM, Malik HS, Emerman M (2008). Antiretroelement activity of APOBEC3H was lost twice in recent human evolution.. Cell Host Microbe.

[25] Betts L, Xiang S, Short SA, Wolfenden R, Carter CW (1994). Cytidine deaminase. The 2.3 A crystal structure of an enzyme: transition-state analog complex.. J Mol Biol.

[26] Johansson E, Mejlhede N, Neuhard J, Larsen S (2002). Crystal structure of the tetrameric cytidine deaminase from Bacillus subtilis at 2.0 A resolution.. Biochemistry.

[27] Ko TP, Lin JJ, Hu CY, Hsu YH, Wang AH (2003). Crystal structure of yeast cytosine deaminase. Insights into enzyme mechanism and evolution.. J Biol Chem.

[28] Xie K, Sowden MP, Dance GS, Torelli AT, Smith HC (2004). The structure of a yeast RNA-editing deaminase provides insight into the fold and function of activation-induced deaminase and APOBEC-1.. Proc Natl Acad Sci USA.

[29] Newman EN, Holmes RK, Craig HM, Klein KC, Lingappa JR (2005). Antiviral function of APOBEC3G can be dissociated from cytidine deaminase activity.. Curr Biol.

[30] Gooch BD, Cullen BR (2008). Functional domain organization of human APOBEC3G.. Virology.

[31] Lei YC, Tian YJ, Ding HH, Wang BJ, Yang Y (2006). N-terminal and C-terminal cytosine deaminase domain of APOBEC3G inhibit hepatitis B virus replication.. World J Gastroenterol.

[32] Janini M, Rogers M, Birx DR, McCutchan FE (2001). Human immunodeficiency virus type 1 DNA sequences genetically damaged by hypermutation are often abundant in patient peripheral blood mononuclear cells and may be generated during near-simultaneous infection and activation of CD4(+) T cells.. J Virol.

[33] Petit V, Guétard D, Renard M, Keriel A, Sitbon M (2008). Murine APOBEC1 is a powerful mutator of retroviral and cellular RNA in vitro and in vivo.. J Mol Biol.

[34] Vartanian JP, Plikat U, Henry M, Mahieux R, Guillemot L (1997). HIV genetic variation is directed and restricted by DNA precursor availability.. J Mol Biol.

[35] Biebricher CK, Eigen M (2005). The error threshold.. Virus Res.

[36] Mansky LM, Temin HM (1995). Lower in vivo mutation rate of human immunodeficiency virus type 1 than that predicted from the fidelity of purified reverse transcriptase.. J Virol.

[37] Vartanian JP, Sommer P, Wain-Hobson S (2003). Death and the retrovirus.. Trends Mol Med.

[38] Bishop KN, Holmes RK, Malim MH (2006). Antiviral potency of APOBEC proteins does not correlate with cytidine deamination.. J Virol.

[39] Nguyen DH, Gummuluru S, Hu J (2007). Deamination-independent inhibition of hepatitis B virus reverse transcription by APOBEC3G.. J Virol.

[40] Suspène R, Henry M, Guillot S, Wain-Hobson S, Vartanian JP (2005). Recovery of APOBEC3-edited human immunodeficiency virus G→A hypermutants by differential DNA denaturation PCR.. J Gen Virol.

[41] Bonvin M, Achermann F, Greeve I, Stroka D, Keogh A (2006). Interferon-inducible expression of APOBEC3 editing enzymes in human hepatocytes and inhibition of hepatitis B virus replication.. Hepatology.

[42] Noguchi C, Hiraga N, Mori N, Tsuge M, Imamura M (2007). Dual effect of APOBEC3G on Hepatitis B virus.. J Gen Virol.

[43] Harris RS, Liddament MT (2004). Retroviral restriction by APOBEC proteins.. Nat Rev Immunol.

[44] Stenglein MD, Matsuo H, Harris RS (2008). Two regions within the amino-terminal half of APOBEC3G cooperate to determine cytoplasmic localization.. J Virol.

[45] OhAinle M, Kerns JA, Malik HS, Emerman M (2006). Adaptive evolution and antiviral activity of the conserved mammalian cytidine deaminase APOBEC3H.. J Virol.

[46] Bishop KN, Holmes RK, Sheehy AM, Davidson NO, Cho SJ (2004). Cytidine deamination of retroviral DNA by diverse APOBEC proteins.. Curr Biol.

[47] Langlois MA, Beale RC, Conticello SG, Neuberger MS (2005). Mutational comparison of the single-domained APOBEC3C and double-domained APOBEC3F/G anti-retroviral cytidine deaminases provides insight into their DNA target site specificities.. Nucleic Acids Res.

[48] Zheng YH, Irwin D, Kurosu T, Tokunaga K, Sata T (2004). Human APOBEC3F is another host factor that blocks human immunodeficiency virus type 1 replication.. J Virol.

[49] Conticello SG, Thomas CJ, Petersen-Mahrt SK, Neuberger MS (2005). Evolution of the AID/APOBEC family of polynucleotide (deoxy)cytidine deaminases.. Mol Biol Evol.

[50] Sawyer SL, Emerman M, Malik HS (2004). Ancient adaptive evolution of the primate antiviral DNA-editing enzyme APOBEC3G.. PLoS Biol.

[51] Xu R, Zhang X, Zhang W, Fang Y, Zheng S (2007). Association of human APOBEC3 cytidine deaminases with the generation of hepatitis virus B x antigen mutants and hepatocellular carcinoma.. Hepatology.

[52] Watts NR, Conway JF, Cheng N, Stahl SJ, Belnap DM (2002). The morphogenic linker peptide of HBV capsid protein forms a mobile array on the interior surface.. EMBO J.

[53] Suspène R, Sommer P, Henry M, Ferris S, Guetard D (2004). APOBEC3G is a single-stranded DNA cytidine deaminase and functions independently of HIV reverse transcriptase.. Nucleic Acids Res.

[54] Benjamin J, Ganser-Pornillos BK, Tivol WF, Sundquist WI, Jensen GJ (2005). Three-dimensional structure of HIV-1 virus-like particles by electron cryotomography.. J Mol Biol.

[55] Xu H, Chertova E, Chen J, Ott DE, Roser JD (2007). Stoichiometry of the antiviral protein APOBEC3G in HIV-1 virions.. Virology.

[56] Holden LG, Prochnow C, Chang YP, Bransteitter R, Chelico L (2008). Crystal structure of the anti-viral APOBEC3G catalytic domain and functional implications.. Nature.

[57] Chiu YL, Soros VB, Kreisberg JF, Stopak K, Yonemoto W (2005). Cellular APOBEC3G restricts HIV-1 infection in resting CD4+ T cells.. Nature.

[58] Chiu YL, Witkowska HE, Hall SC, Santiago M, Soros VB (2006). High-molecular-mass APOBEC3G complexes restrict Alu retrotransposition.. Proc Natl Acad Sci USA.

[59] Petersen-Mahrt SK, Neuberger MS (2003). In vitro deamination of cytosine to uracil in single-stranded DNA by apolipoprotein B editing complex catalytic subunit 1 (APOBEC1).. J Biol Chem.

[60] Larue RS, Andresdottir V, Blanchard Y, Conticello SG, Derse D (2008). Guidelines for Naming Non-Primate APOBEC3 Genes and Proteins.. J Virol.

[61] Larue RS, Jonsson SR, Silverstein KA, Lajoie M, Bertrand D (2008). The artiodactyl APOBEC3 innate immune repertoire shows evidence for a multi-functional domain organization that existed in the ancestor of placental mammals.. BMC Mol Biol.

[62] Kidd JM, Newman TL, Tuzun E, Kaul R, Eichler EE (2007). Population stratification of a common APOBEC gene deletion polymorphism.. PLoS Genet.

[63] Ortiz M, Bleiber G, Martinez R, Kaessmann H, Telenti A (2006). Patterns of evolution of host proteins involved in retroviral pathogenesis.. Retrovirology.

[64] Vartanian JP, Guetard D, Henry M, Wain-Hobson S (2008). Evidence for editing of human papillomavirus DNA by APOBEC3 in benign and precancerous lesions.. Science.

[65] Stunkel W, Bernard HU (1999). The chromatin structure of the long control region of human papillomavirus type 16 represses viral oncoprotein expression.. J Virol.

[66] Endo Y, Marusawa H, Kinoshita K, Morisawa T, Sakurai T (2007). Expression of activation-induced cytidine deaminase in human hepatocytes via NF-kappaB signaling.. Oncogene.

[67] Endo Y, Marusawa H, Kou T, Nakase H, Fujii S (2008). Activation-induced cytidine deaminase links between inflammation and the development of colitis-associated colorectal cancers.. Gastroenterology.

[68] Machida K, Cheng KT, Sung VM, Shimodaira S, Lindsay KL (2004). Hepatitis C virus induces a mutator phenotype: enhanced mutations of immunoglobulin and protooncogenes.. Proc Natl Acad Sci USA.

[69] Matsumoto Y, Marusawa H, Kinoshita K, Endo Y, Kou T (2007). Helicobacter pylori infection triggers aberrant expression of activation-induced cytidine deaminase in gastric epithelium.. Nat Med.

[70] Morisawa T, Marusawa H, Ueda Y, Iwai A, Okazaki IM (2008). Organ-specific profiles of genetic changes in cancers caused by activation-induced cytidine deaminase expression.. Int J Cancer.

[71] Okazaki IM, Hiai H, Kakazu N, Yamada S, Muramatsu M (2003). Constitutive expression of AID leads to tumorigenesis.. J Exp Med.

[72] Yamanaka S, Balestra ME, Ferrell LD, Fan J, Arnold KS (1995). Apolipoprotein B mRNA-editing protein induces hepatocellular carcinoma and dysplasia in transgenic animals.. Proc Natl Acad Sci USA.

[73] Adachi A, Gendelman HE, Koenig S, Folks T, Willey R (1986). Production of acquired immunodeficiency syndrome-associated retrovirus in human and nonhuman cells transfected with an infectious molecular clone.. J Virol.

[74] Thompson JD, Gibson TJ, Plewniak F, Jeanmougin F, Higgins DG (1997). The CLUSTAL_X windows interface: flexible strategies for multiple sequence alignment aided by quality analysis tools.. Nucleic Acids Res.

[75] Kumar S, Tamura K, Nei M (2004). MEGA3: Integrated software for Molecular Evolutionary Genetics Analysis and sequence alignment.. Brief Bioinform.

